# Multimodal investigation of the association between shift work and the brain in a population-based sample of older adults

**DOI:** 10.1038/s41598-022-05418-1

**Published:** 2022-02-22

**Authors:** Nora Bittner, Horst-Werner Korf, Johanna Stumme, Christiane Jockwitz, Susanne Moebus, Börge Schmidt, Nico Dragano, Svenja Caspers

**Affiliations:** 1 Institute for Anatomy I, Medical Faculty and University Hospital Duesseldorf, Heinrich-Heine-University, Universitaetsstraße 1, 40225 Duesseldorf, Germany; 2 Institute of Neuroscience and Medicine (INM-1), Research Centre Juelich, 52428 Juelich, Germany; 3 Institute of Urban Public Health, University of Duisburg-Essen, 45122 Essen, Germany; 4 Institute for Medical Informatics, Biometry and Epidemiology, University Hospital of Essen, University Duisburg-Essen, 45130 Essen, Germany; 5 Institute of Medical Sociology, Medical Faculty, University of Düsseldorf, 40225 Düsseldorf, Germany; 6 JARA-BRAIN, Juelich-Aachen Research Alliance, 52427 Juelich, Germany

**Keywords:** Neuroscience, Circadian rhythms and sleep, Cognitive neuroscience

## Abstract

Neuropsychological studies reported that shift workers show reduced cognitive performance and circadian dysfunctions which may impact structural and functional brain networks. Here we tested the hypothesis whether night shift work is associated with resting-state functional connectivity (RSFC), cortical thickness and gray matter volume in participants of the 1000BRAINS study for whom information on night shift work and imaging data were available. 13 PRESENT and 89 FORMER night shift workers as well as 430 control participants who had never worked in shift (NEVER) met these criteria and were included in our study. No associations between night shift work, three graph-theoretical measures of RSFC of 7 functional brain networks and brain morphology were found after multiple comparison correction. Preceding multiple comparison correction, our results hinted at an association between more years of shift work and higher segregation of the visual network in PRESENT shift workers and between shift work experience and lower gray matter volume of the left thalamus. Extensive neuropsychological investigations supplementing objective imaging methodology did not reveal an association between night shift work and cognition after multiple comparison correction. Our pilot study suggests that night shift work does not elicit general alterations in brain networks and affects the brain only to a limited extent. These results now need to be corroborated in studies with larger numbers of participants.

## Introduction

Shift work is a major challenge for the human circadian system and especially night shift work can provoke a conflict between the exogenous work schedule demands and an individual’s circadian rhythms defined as chronotype. Dysfunctions of the circadian system may be associated with lower mental and physical health^1^. Several previous studies focused on the relationship between shift work and cognition, but they have provided inconsistent and variable results^2,3^. A prospective cohort study led to the conclusion that “*shift work chronically impairs cognition, with potentially important safety consequences not only for the individuals concerned, but also for society*”^4^. This study has received ample attention by the press media (Shift work dulls your brain, BBC News, 4 November 2014; Long term shifts ages brains, Sky News, 4 November 2014 https://www.nhs.uk/news/neurology/shift-work-ages-the-brain-study-suggests/). However, Machi, et al.^5^ investigated early carrier physicians and reported a decline in short-term memory after day and over nightshifts and a high incidence of disturbed sleep, while in another study cognitive flexibility during night shifts was not altered per se, but largely depended on the circadian phase of the individual^6^. No difference in late-life cognitive aging was observed between individuals with a history of working shifts as compared to those who had typical day work schedules during midlife^7^. Additionally, Titova, et al.^8^ showed altered performance in present, but not former shift workers.

It has been proposed that cognitive impairment in shift workers may be a consequence of neuronal disruptions, such as malfunctioning of brain regions involved in circadian rhythms^4^. Circadian misalignment has indeed been discussed to impact on neuronal pacemakers^1^ and to play a role in psychiatric disorders^9^. Further, the individual chronotype, i.e. the intrinsic, biological preference for an early or late sleep onset, has recently been shown to modulate functional connectivity (FC) of the large-scale default mode brain network involved in cognitive functions^10^. The individual chronotype may also have an impact on the ability to cope with shift work^11,12^, therefore constituting a potentially important influence. Alterations within neuronal networks associated with shift work may therefore be one explanation for cognitive performance differences.

Previous studies have shown a high variability in cognitive abilities in older adults^13–15^ which may be influenced up to old ages by various factors^16–19^, such as education and lifestyle^20,21^. Here we tested whether shift-work is related to neuronal differences and therefore another factor for accelerated brain and cognitive aging utilizing the population-based 1000BRAINS study which was designed to examine the variability of brain phenotypes during the course of aging with regard to influencing factors.

To this end we analyzed resting state functional connectivity (RSFC) derived from magnetic resonance imaging (MRI) since a previous study showed that the individual chronotype was related to RSFC of the default mode network^10^. RSFC has been used as a marker for general functional brain architecture and intrinsic communication^22,23^. It is involved in cognitive abilities, which has not only been shown for higher-order cognitive networks (e.g. fronto-parietal, ventral and dorsal attention networks), but also for primary processing networks^24^ such as the visual and sensorimotor network. Moreover, cognitive performance differences seem to largely depend on the communication and cooperation *within* these functional networks, as well *between* these functional networks^24,25^. This allows hence to investigate, e.g. why some older adults experience greater cognitive decline than others. A highly segregated network, i.e. showing high within-network RSFC, is thought to be particularly specialized and effective, while highly integrated networks largely depend on other networks and are thought to be reduced in their specificity. More segregated networks may also constitute a more resilient functional state against certain types of changes such as aging, neurodegenerative disease^26,27^ or circadian disruption through shift work. Further, the explanatory power of network-wise RSFC for cognitive performance has already been shown within a subsample of the here investigated 1000BRAINS cohort^24^. We therefore chose to test for differences in within- as well as between-network RSFC and as well as the relation between integration and segregation.

Importantly, cognitive performance as a complex, higher-order brain function comprises several brain structural correlates, particularly within the cortex^28–30^. An extensive body of research established the relationship between cortical thickness and cognitive performance^31^ in adolescents^28,32^, younger and older adults^33^ as well as in patients suffering from neurodegenerative disorders^34,35^. Further, cortical thinning has been proposed as a surrogate marker for the early diagnosis of Alzheimer's disease^34^. Cognitive decline in ageing and neurodegenerative diseases further affects subcortical structures, such as the hippocampus^36–38^. A previous study addressed the problem whether jetlag in flight attendants with short and long recovery periods is associated to volume reduction of the right temporal lobe^39^. In those with short recovery periods a correlation was found between saliva cortisol levels, lower volume of the right temporal lobe and longer reaction times in a visual-spatial memory task. Taking these previous results into consideration we tested for shift work related differences in cortical thickness across the whole cortical surface, as well as for differences within subcortical gray matter including the hippocampus.

The objective brain investigations were supplemented by a large set of neuropsychological examination indicative for performance in several cognitive domains. Based on previous literature, we paid particular attention to the domains of attention, short-term and working memory, processing speed^8,40^, as well as executive functions^41,42^. Aiming for a complete examination, we also employed tests shown to be sensible to age-related decline in cognitive domains^13–15^ including visual-spatial memory, vocabulary, creative thinking and reasoning^13,43^.

To test the hypothesis whether night shift work is associated to differences in brain parameters and cognitive performance, we addressed three questions:

1. Do present shift workers show differences in brain parameters in comparison to controls? We therefore compared NEVER shift workers with PRESENT shift workers regarding (i) RSFC, (ii), cortical thickness and (iii) volume of subcortical structures. Concurrently, both groups were compared regarding their cognitive performance.

2. Do brain parameters (neuronal correlates of cognition) differ between previous shift workers and non-shift workers? This question related to the problem whether the observed differences may be reversible^4^. Hence, we compared NEVER shift workers with FORMER shift workers regarding brain parameters, as well as cognitive performances.

3. Is a longer employment in shift work (measured in number of shift work years) associated to a stronger alteration in brain parameters, accompanied by lower cognitive performance? We supplemented this by correlation and mediation analyses to establish the triangular association between shift work, differences in brain parameters and cognitive performance.

## Materials and methods

### Participants

Data were collected from participants of the 1000BRAINS study^43^, recruited from the Heinz Nixdorf Recall study^44^. The study was approved by the Ethics Committee of the University of Essen (Germany). All participants gave written informed consent in agreement with the declaration of Helsinki prior to participation. To test the hypothesis whether night shift work is associated with resting-state functional connectivity, cortical thickness and gray matter volume 532 participants (287 men and 245 women) of the 1000BRAINS study for whom information on night shift work and imaging data were available were included in this study.

Shift work parameters were obtained in an interview in which the participants were asked whether they worked in shift at any time of their life (“Yes”/“Never”), with shift being defined as a work schedule outside the period between 7am to 6 pm. Participants who answered “Never” served as control group. Participants who answered “Yes” were asked (i) which shift schedule they were engaged in (rotating shifts without night shifts, rotating shifts including night shifts or early shifts, late shifts and night shifts only), (ii) how many years they worked in shift, and (iii) whether they worked in shift at time of data acquisition. The present study includes participants who worked either in night shifts only or in rotating shifts including night shifts, since night shifts are the greatest challenge for the human circadian system and therefore have the greatest impact on health parameters^1^.

According to the shift work status, participants were divided into three groups. The first group comprised participants, who stated that they had never performed shift work, and constitutes the control group (NEVER shift workers, n = 430, 207 male, 223 females). The second group comprised participants, who worked in night shift or rotating shifts including night shift at the time of data acquisition or within the last year before. This group is defined as PRESENT shift workers and comprised 13 participants (9 males, 4 females). Two participants worked in night shifts only, the remaining 11 worked in rotating shift systems, which included night shifts. The third group comprised 89 participants who had stopped night shifts two or more years before the time point of data acquisition and is defined as FORMER shift workers (71 males, 18 females). Ten participants had worked only in night shift, while all others had worked in rotating shift systems, including night shifts. Characteristics of these groups are depicted in Table 1.

**Table 1:** Descriptive group statistics.

Variable	PRESENT (n = 13)/MATCHED controls (n = 13)	FORMER (n = 89)/MATCHED controls	NEVER (n = 430)
Age (years)	60.99 (SD = 2.27)/61.22 (SD = 3.90)	67.70 (SD = 6.40)/68.33 (SD = 6.49)	67.06 (SD = 6.49)
Sex	9 males, 4 females/9 males, 4 females	71 males, 18 females/74 males, 13 females	207 males, 223 females
Education	5.92 (SD = 1.89)/5.38 (SD = 0.77)	6.38 (SD = 1.96)/6.29 (SD = 1.90)	6.35 (SD = 1.94)
Smoking (Pack-years)	29.27 (SD = 23.88)/9.22 (SD = 14.29), *p* = 0.006	20.42 (SD = 30.71)/17.28 (SD = 21.48)	12.31 (SD = 18.78)
Alcohol consumption	96.57 (SD = 120.95)/68.77 (SD = 107.37)	86.82 (SD = 108.15)/85.65 (SD = 172.68)	70.21 (SD = 130.77)
Coffee Consumption	4.50 (SD = 1.00)/4.69 (SD = 0.86)	4.46 (SD = 1.21)/4.48 (SD = 1.19)	4.55 (SD = 1.08)
Black Tea consumption	2.25 (SD = 1.36)/1.77 (SD = 0.93)	2.10 (SD = 1.34)/1.93 (SD = 1.28)	2.10 (SD = 1.43)
Shift work years	19.77 (SD = 12.11)/0	10.07 (SD = 10.06)/0	0

Group statistics are given in unadjusted means (Standard deviation) for purposes of interpretability. Alcohol consumption was measured in grams of pure alcohol per week. For the ordinally scaled variables of coffee and black tea consumption the following scale was used: 1.00 = Almost never, 2 = 1–3 times per month, 3 = 1–3 times a week, 4 = 4–6 times a week; 5 = daily.

### Imaging

To test whether shift work is associated with measurable, objective differences in functional connectivity and morphology imaging data were analyzed which were collected using a 3T Siemens Tim-Trio MR scanner with a 32-channel head coil (Erlangen, Germany) and different MR sequences. For the surface reconstruction, cortical thickness and subcortical gray matter volumes analyses, a 3D high-resolution T1-weighted magnetization-prepared rapid acquisition gradient-echo (MP-RAGE) anatomical scan was acquired with 176 slices (slice thickness 1 mm, repetition time (TR) = 2250 ms, echo time (TE) = 3.03 ms, field of view (FoV) = 256 × 256 mm^2^, flip angle = 9°, voxel resolution 1 mm^3^) lasting about 5 min. Resting-state functional MRI measurements were performed using a blood-oxygen level dependent (BOLD) sequence with 36 transversally oriented slices, measured using a gradient-echo echo planar imaging (EPI) sequence (slice thickness 3.1 mm, TR = 2200 ms, TE = 30 ms, FoV = 200 × 200 mm, voxel resolution 3.1 mm^3^) for about 11 min, resulting in 300 volumes. During this sequence, participants were instructed to keep their eyes closed, be relaxed, let their mind wander, and not fall asleep. The latter was secured by post-scan debriefing (for a detailed description of the 1000BRAINS study protocol, see Caspers et al.^43^).

### Preprocessing of Resting-State functional images

Preprocessing of resting-state data was performed using FSL [FMRIB Software Library: http://www.fmrib.ox.ac.uk/fsl^45^]. Participants’ functional images were motion corrected and co-registered to the individual anatomical scan using FMRIB´s Linear Image Registration tool [MCFLIRT and FLIRT^46^]. Then, slice time correction [slicetimer^47^], brain extraction [BET^48^], intensity normalization and spatial smoothing (5 mm at FWHM) [SUSAN^49^] was performed. Data-driven identification and removal of motion-related components from functional MRI data [ICA-based Automatic Removal of Motion Artifacts; ICA-AROMA^50^] was done. Further, global signal regression^51–53^ as well as bandpass filtering (0.01–0.1 Hz) was applied. Then all functional images were registered to the standard space template of MNI 152 using FSLs nonlinear registration tool [FNIRT^54^].

The “check sample homogeneity using standard deviation across sample” analysis provided by the Computational Anatomy Toolbox [CAT12^55^] was used to check whether individual images matched the MNI152 template. All participants included in this study were manually checked to control for possible outliers. Volume-wise severe intensity dropouts were checked for each participant by generating *p* values for spikes (DVARS) on the already preprocessed functional data as established by Afyouni and Nichols^56^.

### Resting-State functional connectivity

To investigate resting state functional connectivity (RSFC) in large scale brain networks, which have recently been found to be sensible to circadian rhythmic and individual chronotype^10^, we used the cortical parcellation of^57^. This parcellation scheme was established based on intrinsic RSFC from 500 participants (checked with a 500 subjects replication cohort). Whole-brain RSFC was clustered into 7 networks based on their similarity of functional activation over all participants. Similarity of functional activation is here defined by time-wise coactivation between spatially distributed regions. Thus, regions, which are likely coactivated, belong to the same functional network. The resulting 7-network parcellation mainly distinguishes known functional RS networks, namely visual- (VN), sensorimotor- (SMN), limbic- (LIMN), frontoparietal- (FPN/control network), default mode- (DMN), dorsal (DAN)- and ventral attention (VAN) network (Fig. 2A). The 7 networks comprise 400 parcels in total, each of which can be allocated to one network, such that one network comprises several different parcels (i.e. regions). Interindividual variance within the cluster of parcels (i.e. networks) due to transformation from subject to standard space was addressed by eroding using FSL [fslmaths -ero^58^]. Voxels with less confidence of network affiliation were discarded as a consequence.

The association between shift work and network-wise RSFC was investigated using graph-theoretical parameters^24,59^. Therefore, a whole brain graph (i.e. connectome, Rubinov and Sporns^60^) was built based on individual functional data. Here, each parcel was defined as a node. Each node was reflected by a BOLD mean time series spanning 300 time points, i.e. the time series of all voxels corresponding to that node (i.e. to that region) were averaged based on the preprocessed RS-fMRI data [fslmeants^58^]. Edges were then defined as the functional connection between two nodes, which was calculated using FFT Permutation testing^61^ of the respective BOLD mean time series of the two nodes. Based on the 400 parcels, 400 × 400 FFT correlation coefficients were determined, each reflecting the functional connection between two nodes (i.e. between two regions). Using Fishers r-to-z transformation these coefficients were transformed into z-scores containing both positive and negative correlations. Since the integration of positive and negative weights into the estimation of strength values may possibly lead to a mutual suppression, we only performed estimations with positive correlations.

In RSFC the calculation of functional connections is based on correlations between minimal BOLD activity fluctuations, leaving a risk of measuring noise rather than of true signal. To improve the signal to noise ratio, the statistical significance of each correlation coefficient was tested by randomizing the observed timeseries by taking its Fourier transform, scrambling its phase and then inverting the transform^61^. After repeating this procedure 1000 times, a permutation test was applied and non-significant edges at *p* > 0.05 were discarded. As a consequence, networks may consist of inter-individually different amounts of edges. To ensure that comparisons between participants would not be distorted by these varying amounts of edges, we focused on the strength value as a reliable network parameter, robust against this issue^62^. For a more detailed discussion of this topic see Stumme et al.^24^.

The software bctpy with network parameters as defined in Rubinov and Sporns^60^ was used to quantify the RSFC of networks. Strength values were computed for each node as the sum of connectivity weights attached to that node. Based on these strength values, three different RSFC parameters were calculated for each of the seven networks described above. We calculated composite within- and inter-network RSFC for each participant, to limit the number of pairwise comparisons:

(i) Within-network RSFC was computed as the mean connectivity of edges between all pairs of nodes belonging to the same network. The sum of all edge weights of all nodes within a network was calculated and divided by the number of edges in that network, thus accounting for individually varying number of nodes.

(ii) Inter-network RSFC was computed as the sum of connectivity of edges from each node within the network to all nodes outside the network, divided by the total number of edges.

(iii) Between-network RSFC was computed as the sum of all edges between pairs of nodes between two specific networks, divided by the number of edges belonging to both networks.

Additionally, a combined quantitative ratio was determined to capture the within-network RSFC in relation to the inter-network RSFC:

$$\frac{within{-}network\,RSFC-inter{-}network\,RSFC}{within{-}network\,RSFC+inter{-}network\,RSFC}$$

Using this ratio score, which was employed by Chan, et al.^25^ and refined by Stumme, et al.^24^ the network’s segregation can be quantified. Specifically, a ratio-score of 1 implies maximal network segregation (high within- and low inter-network RSFC), whereas a ratio-score of − 1 indicates maximal network integration (low within- and high inter-network RSFC). A score of zero indicates a balanced system.

### Preprocessing of structural imaging data

The 3D anatomical images were processed using the automated surface-based pipeline of the FreeSurfer Software package^63^ (version 6.0, Athinoula A. Martinos Center for Biomedical Imaging). A detailed description of all steps included in the streamline was provided by Dale et al.^64^ and in the FreeSurfer documentation at http://surfer.nmr.mgh.harvard.edu. First, segmentation into gray matter (GM), white matter (WM) and cerebrospinal fluid (CSF), motion correction, intensity normalization and removing of extra-cerebral voxels (non-brain tissue) was done using CAT12^55^. The resulting preprocessed volumes were fed into the default surface-reconstruction pipeline “recon-all” of FreeSurfer, where transformation into Talairach space, the tessellation of GM/WM boundary, cortical surface reconstruction^64,65^ and correction of topological defects was performed. To reconstruct the cortical surface, first the so-called “white” surface was generated at the interface of WM and GM. Then, the pial surface was created at the interface between GM and CSF. The final mesh model of the pial surface is tessellated into triangles and consists of about 120,000 vertices per hemisphere with an average surface area of 0.5 mm^2^. Cortical thickness (CT) was then measured by finding the shortest distance between a given vertex on the reconstructed pial surface and the respective corresponding vertex on the GM/WM boundary (“white”) surface and vice versa^66^. Finally, averaging both values resulted in about 120,000 CT values per hemisphere. For each vertex, the cortical thickness can then be related to influencing variables, such as shift work.

Subcortical structures were segmented using the automatic segmentation provided by FreeSurfer^67^ as well. Here, subcortical GM is automatically segmented into different volumes. Then, a neuroanatomical label is assigned to each volume based on probabilistic information estimated from a manually labeled data set. Subcortical volumes comprised the thalamus, caudate, putamen, pallidum, hippocampus, amygdala, and accumbens nucleus, bilaterally^67^. Total subcortical and total GM volume were examined.

### Neuropsychological performance

To assess whether night shift work affects cognitive performance we selected several neuropsychological tests from the large battery provided by the protocol of the 1000BRAINS study^43^. To cover the main domains and to include tests that represent cognitive abilities which have previously been associated with shift work^4^ we particularly focused on the domains of attention, working memory, processing speed and executive function. The domain of selective attention was covered by the “Aufmerksamkeits-Konzentrations-Test” [*AKT (Time)*] in which the time participants needed to cross out target figures from similar distractor figures, was measured^68^. Working memory was investigated using a non-verbal, as well as a verbal working memory test: Non-verbal (spatial) working memory was assessed using the Corsi block-tapping test (CBT)^69^ in which the participants needed to reproduce a sequence of blocks (increasing from 2 to 9 blocks each trial) on a board of 9 blocks in equal [*CBT (Forward)*] or reverse order [*CBT (Backward)*]. Number of maximal correctly reproduced blocks was measured. To address verbal working memory, we chose the verbal equivalent, the Zahlen-Nachsprechen-Task [ZNS; from Nürnberger Alters-Inventar^70^]. Here, a digit span is read to the participant with complexity increasing from 2 to 9 digits in each trial. The maximal digit span was measured, which the participant was able to reproduce in equal [*ZNS (Forward)*] as well as reverse order [*ZNS (Backward)*]. Further, visual working memory was tested using the Visual pattern (Jülich version; similar to: Della Sala et al., 1997) test, where the total number of correctly memorized matrix patterns of black and white squares with increasing complexity was measured. Processing speed was assessed using the Trail-Making-Test [taken from CERAD-Plus;^40^]. Task A (*TMTA*) measures the time, the participants need to connect randomly arranged digits printed on a piece of paper in ascending order as fast as possible. Task B (*TMTB*) measures the same, but the participants need to connect digits and letters alternatingly in ascending order, which invokes task-switching processes between the concept of letters and of digits. Additionally, the time difference between task A and B was calculated [*TMTBA (Switching)*], reflecting the cost function for the higher cognitive demand of task B, which is indicative for concept shifting performance in the domain of executive function. The second task reflecting executive function was given by the German “Farb-Wort-Interferenz-Test”, similar to the Stroop test [Jülich version; similar to^41,71^]. In the first step [*Stroop (Reading)*], time needed to read color words printed in black ink as fast as possible was taken. In the second step [*Stroop (Naming)*], time needed to name the color of colored squares was measured. Third, the participants were presented with color words printed in a different color than the color word refers to [*Stroop (Selectivity)*]. Time needed to name the color in which the color word is printed was taken. While the first two tasks were examined as measures of processing speed, the last task involves the process of inhibition, which is also called interference of tasks. The cost function for this interference task (Task 3 minus Task 1), reflects the ability to inhibit automatic processes and therefore executive performance [*Stroop (Interference)*].

Furthermore, we analyzed cognitive tests employed by the 1000RBAINS protocol, which have been rarely employed in studies of shift work, e.g. the Wortschatztest^72^ measuring vocabulary, i.e. the total number of correctly identified real words within rows of pseudo words. Figural fluency/creativity was examined with the Fünf-Punkte-Test (Jülich version; similar to:^73^) measuring the total number of unique designs created by connecting 5 dots (3 min). Figural memory was examined using the Benton test^74^, examining the total number of errors made during the free recall of 20 previously presented figures. Finally, another domain assessed in 1000BRAINS was reasoning (Leistungsprüfungssystem 50 + (Subtest 3)^75^, where irregularities in serials of geometric figures needed to be tagged (5 min). In total this resulted in a large battery of cognitive parameters belonging to 12 neuropsychological tests, such that we accepted one missing value per participant for cognitive analyses.

### Chronotype

The chronotype of each participant was determined by calculating the mid sleep on free days (MSF), a value introduced by Roenneberg et al.^76^ based on the participants´ answers to the questions when they would get up and go to bed if they were able to design their day freely according to their own comfort. MSF is the midpoint between wake-up and go-to-bed time and is given in hours (h) and minutes (min).

### Sample collection and statistical analyses

For all four domains investigated in the present study, (1) RSFC, (2a) morphology of cortical thickness, (2b) morphology of gray matter subcortical volume, and (3) cognitive performance, the same analysis procedure was employed, which will be described in the following: We first examined a main effect of shift work, then we compared the shift work groups to MATCHED samples and finally we compared shift work groups to RANDOM samples.

### Main effect of shift work

To evaluate whether shift work has a general impact on brain function, structure and cognitive performance we first compared the two shift working groups (PRESENT and FORMER) to the whole control group of NEVER shift workers. To this end, we evaluated the main effects of *shift work group* in two multivariate between-subjects-analyses of variance (ANOVA) with the independent factors sex (male, female) and shift work group (PRESENT, FORMER, NEVER) as well as age and education as covariates. Education was defined by the international standard classification of education (ISCED)^77^. The first ANOVA was set up as a multivariate ANOVA and used all RSFC-parameters as dependent variables. The second MANOVA used all subcortical volumes as dependent variables. To evaluate cognitive performances, we employed several univariate ANOVAS examining each cognitive task as dependent variable respectively, using the same setup with independent factors sex (male, female) and shift work group (PRESENT, FORMER, NEVER) as well age and education as covariates. This was done since some participants lacked data in one of the cognitive tests. Calculating one multivariate analysis of variance using all cognitive tests as dependent variables would have further reduced the sample size of the PRESENT shift workers. To evaluate effect sizes partial eta squared was used.

Since the group of NEVER shift workers was much larger (n = 430) than the groups of PRESENT shift workers (n = 13) or FORMER shift workers (n = 89), the statistical power for direct group comparisons was rather small for a precise estimation of the effect size, particularly within PRESENT shift workers.

We therefore implemented two different approaches for group comparisons.

### Random samples

To take advantage of our rich sample of control participants (NEVER shift workers) we first selected 1000 random samples of the 430 control participants (RANDOM controls) and compared these with the two groups of PRESENT and FORMER shift workers, independently. This approach was motivated by the advantage that bootstrap techniques offer in light of small sample sizes^78,79^. Here, in comparison to finding matching controls, controls are randomly drawn from the population to resample how a randomly resampled group of controls can be compared to the shift workers. Random samples were drawn using the function “randomsamples” (“Zufallsstichprobe”) implemented in R [^80^; https://www.R-project.org/], which was then repeated 1,000 times. For each iteration of drawing a random sample, all NEVER shift workers were available (sampling with replacement). For each of the random samples (RANDOM controls), mean values of all 21 RSFC parameters and mean performance in the 12 cognitive tests were taken, which were than compared to the means for the respective variable of the PRESENT as well as the FORMER shift worker group. Means of the PRESENT shift workers were tested against each random sample using Mann–Whitney-U-tests with an alpha-level of α = 0.05 (two-tailed). Means of the FORMER shift worker group were tested against each random sample using Analysis of variance (ANOVA), corrected for age, sex and education, using the same alpha-level (two-tailed). The percentage of comparisons out of 1,000 showing a significant difference between the random samples and the samples from both PRESENT shift workers and FORMER shift workers was taken. An alpha level of 0.05 was generally considered significant, i.e. more than 95% of the 1000 comparison between RANDOM controls and PRESENT or FORMER shift workers, respectively, needed to show a significant difference. We applied an additional correction for multiple comparisons within each domain (please see “Correction for multiple comparisons”). The alpha level considered significant for the RSFC parameters was *p*RSFC_corr_ = 0.002381, such that 99.8% of the comparisons needed to be significant. For cognitive parameters, the alpha level was *p*COGNITION_corr_ = 0.01 and hence 99.0% of the comparisons needed to be significant. If Mann–Whitney-U-tests (NEVER versus PRESENT) or ANOVA (NEVER versus FORMER) reached the respective statistical threshold, we determined whether the random sample of NEVER shift workers, PRESENT shift workers or FORMER shift workers had higher mean scores. For comparison between NEVER and PRESENT shift workers we chose Mann–Whitney-U-tests as the non-parametric equivalent to t-tests due to the small sample size and calculated Pearson’s correlation coefficient as $r=\frac{Z}{\sqrt{n}}$ using the standardized test statistic *Z* and the sample size *n*. For all analyses of variance partial eta squared as calculated within SPSS was used as effect size.

### Matched samples

In a second more clinically motivated approach, we defined matched groups for each of the two shift working groups: From the 430 NEVER shift workers we selected participants comparable in age, sex and education. Matching was done by propensity score matching using the “match-it”-algorithm^81,82^ implemented in R for the two shift working groups independently. Hence, 13 matching partners were found for the group of PRESENT shift workers. For the group of 89 FORMER shift workers, 87 matching partners were found.

For each matched pair of groups (NEVER versus PRESENT, NEVER versus FORMER) we compared the mean of the shift working group for each variable against the mean of the matched control group using non-parametric Mann–Whitney-U tests (two-tailed, α = 0.05). We chose non-parametric tests since the sample size of PRESENT shift workers was limited and network-wise RSFC as well as cognitive performance data was not normally distributed. Pearson´s correlation coefficient was calculated as effect size as described above.

### Group comparisons of cortical thickness

To examine possible associations between shift work and the structure of specific cortical regions we chose a vertex-wise analysis along the whole cortical surface. Since it was not possible to draw 1,000 random samples of NEVER shift workers within FreeSurfer and to compare them against PRESENT or FORMER shift workers, we took a different approach to investigate possible associations between shift work and regional variations in cortical thickness.

First, we carried out two univariate general linear models, as implemented in QDEC, a graphical user interface provided by FreeSurfer^63^. First, PRESENT shift workers were compared to all NEVER and then FORMER shift workers were compared to all NEVER shift workers, both times correcting for age, sex and education.

For the MATCHED analysis, we compared pairs of matched controls of NEVER and PRESENT and NEVER and FORMER shift workers against each by means of QDEC, a graphical user interface implemented in FreeSurfer using general linear models. Group was given as a factor, and age, sex and education as covariates, while vertex-wise cortical thickness was the dependent variable. We defined a cluster-forming threshold of α = 0.001 (two-tailed) and corrected for multiple comparisons using Monte Carlo Null distributions with α = 0.05. The threshold of α = 0.001 was chosen since it corresponds more closely to a type-1-error-probability of 5%^83^.

Second, we tested for an association between years of shift as an explanatory variable and vertex-wise cortical thickness as the dependent variable within the general linear model, while introducing age, sex and education as covariates. This was done independently for PRESENT and FORMER shift workers.

### Linear association with the number of shift years

To determine whether the number of shift years was associated with alterations of any parameter investigated here, we calculated multiple linear regression analyses within IBM SPSS Statistics 26 (https://www.ibm.com/de-de/analytics/spss-statistics-software) using age, sex and educational level (covariates) and the number of shift years as explanatory variables and (i) all network-wise RSFC parameters, as well as (ii) gray matter volume of subcortical structures, and (iii) all cognitive performance scores using two-tailed tests. Network-wise RSFC parameters mostly resampled a normal distribution, whereas cognitive performance scores deviated more from normal distribution. There was no general shift to one side of the Gaussian curve, and therefore general transformation of all cognitive scores was no solution to this issue. Since it is mostly agreed that linear regression can be used despite non-normality^84,85^ and our approach is exploratory, we continued calculating linear regressions.

### Mediation analyses

We hypothesized that the association between shift work and cognitive performance may be driven by neuronal differences. So far, we examined the association between shift work group and number of shift work years with RSFC *or* brain morphology (cortical thickness, gray matter volumes) *or* cognitive performance independently. We additionally examined the triangular association between (i) shift work, (ii) RSFC or brain morphology and (iii) cognitive performance. To this end, partial correlations corrected for age, sex and education between all RSFC parameters and cognitive performances were calculated.

**Figure 1:**
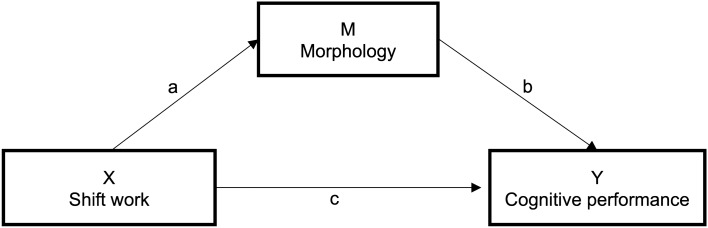
Representation of the mediation analyses, were the triangular association between Shift work (x), brain Morphology as a mediator (m) and Cognitive performance as outcome (y) is tested. Arrows “a” and b” via “M” represent the indirect effect of shift work via M on cognitive performance, while “c” describes a direct association.

We further employed mediation analyses to investigate whether the link between (X) shift work and (Y) cognitive performance may be mediated by (M) brain morphology (Fig. 1).

Mediation analyses were only calculated for those cognitive performances, which showed a significant association (before Bonferroni correction) to shift work, as this link is a prerequisite, that a mediation effect can be present.

### Group differences

The first series of mediation analyses were calculated with shift work group (PRESENT, FORMER, NEVER) as explanatory factor (X).

Cognitive performance that differed between shift work groups were working memory [digit span, i.e. ZNS (Forward)], processing speed [Stroop (Naming); Stroop (Reading)] (ANOVAS) as well as processing speed (TMT-A), and concept shifting (TMT-BA) (MATCHED comparisons) and therefore used as outcomes (Y) for all mediation analyses.

Regarding morphology, no association between shift work and cortical thickness was found. For subcortical volumes, ANOVAS indicated an association between shift work and the left thalamus. Hence, gray matter volume of the left thalamus was entered as mediator within the first series of mediation analyses replacing RSFC of the visual network.

### Shift work years

The next series of mediation analyses used the number of shift work years as explanatory factor (X). Since no association between years of shift work (X) and cognitive performance (Y) was found in PRESENT shift workers, the prerequisite that a direct association between X and Y is given was not fulfilled and no mediation analyses were calculated.

In FORMER shift workers, an association between shift work years and performances in selective attention (AKT), reasoning, processing speed (Stroop) and susceptibility for interference [Stroop (interference)] were found and therefore used as outcomes (Y) for the second series of mediation analyses. Gray matter volume of the left thalamus was used as mediator (M).

### Multiple comparison correction

Between NEVER and PRESENT and between NEVER and FORMER shift workers 21 group comparisons and 21 regressions for network-wise RSFC parameters were calculated. This would have led to a Bonferroni correction of 0.05/21 = 0.002381 in each group.

For all analyses regarding volume of 7 subcortical structures within each hemisphere we used a Bonferroni correction of 0.05/14 = 0.0036 in each group.

Further, since all cognitive parameters belong to 12 independent tests, a Bonferroni correction led to a corrected *p* value of 0.05/12 = 0.004. For cortical thickness, NEVER were compared to PRESENT and to FORMER shift workers for both hemispheres of the brain and a corrected *p*-value of *p* = 0.001 was applied (as described above).

However, regarding the small sample size of the PRESENT shift working group it is not to be expected that any association would reach this significance level. We therefore compare the results with the Bonferroni corrected p-values to give a statistical guideline. We also still discuss results, which did not reach this significance threshold and focus to this end on effect sizes to further guide the interpretation of the results. We consider this appropriate since the reliance on effect sizes and confidence intervals gains more importance within cognitive psychology in comparison to reliance on *p* values^86–88^.

## Results

### Descriptive statistics

Control participants, i.e. NEVER shift workers were on average 67 years old. PRESENT shift workers were on average 61 years old, FORMER shift workers were on average 68 years old. There were no differences between the groups in terms of lifestyle, except for PRESENT shift workers showing significantly higher pack-years of smoking than MATCHED controls (Table 1).

### Resting-state functional connectivity

#### Analysis of variance

**Figure 2:**
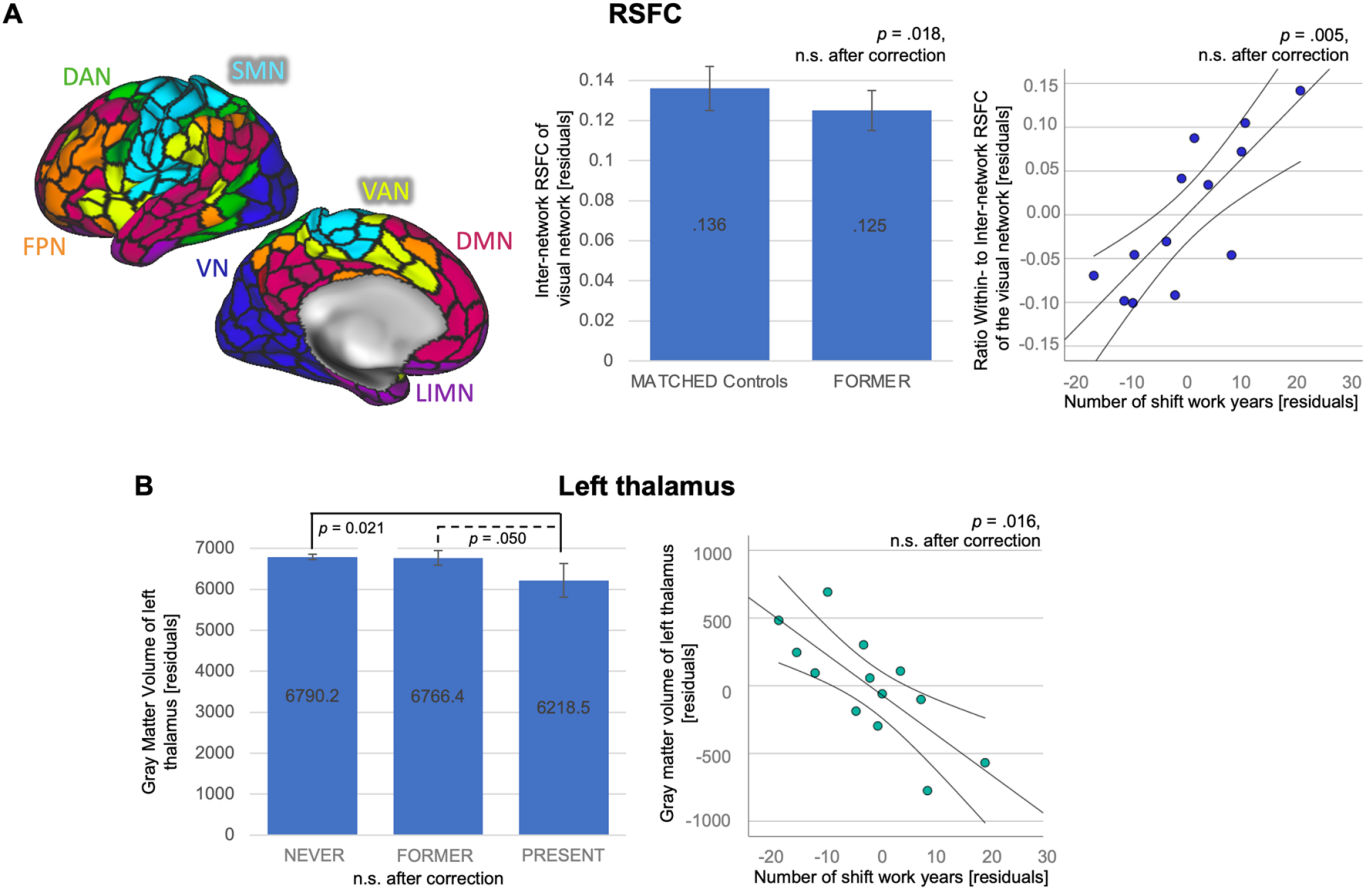
Imaging analyses. (**A**) Representation of the 7 functional networks on the left lateral surface of the brain: visual network (VN), dorsal attention network (DAN), ventral attention network (VAN), sensori-motor network (SMN), fronto-parietal network (FPN), limbic network (LIMN) and default mode network (DMN). FORMER shift workers showed lower *inter*-network RSFC of the visual network than MATCHED controls, while a higher number of shift work years was associated with a higher ratio of *within*- to *inter*-network connectivity of the visual network in PRESENT shift workers (n = 13). (**B**) PRESENT shift workers showed lower gray matter volume of the left thalamus compared to FORMER and all NEVER shift workers in multivariate analysis of variance, corrected for age, sex, education and total gray matter volume. 95% confidence intervals are indicated by lines surrounding the regression lines and are given in detail in Table 3 for the regression coefficients. Parameters are represented in residuals from partial correlations. None of these association were significant after multiple comparison correction.

To examine a significant main effect of shift work on RSFC a multivariate analysis of variance (ANOVA) was employed. We used sex (male, female) and shift work group (NEVER, PRESENT, FORMER) as independent factors, age and education as covariates and the 21 RSFC parameter as dependent variables, i.e. within-network, inter-network connectivity and the ratio of *within- to inter-network* connectivity of each of the 7 large-scale cortical brain networks (Fig. 2).

Here, no main effect of shift work on RSFC was found [F (42, 1010) = 1.10, *p* = 0.309, Wilks Λ = 0.914; partial *η*^2^ = 0.044].

### Matched samples comparisons

There were marginally significant differences in RSFC parameters (Table 2) for *within-network* RSFC of the fronto-parietal network (FPN, *p* = 0.034) and the ratio of *within- to inter-network* RSFC of the visual network (*p* = 0.044; Fig. 2) between PRESENT shift workers and MATCHED controls.

**Table 2:** Results on network-wise Resting State Functional Connectivity (RSFC).

Compared to	RANDOM	MATCHED		Linear regression of shift work years			
	Sign (%)	Mean rank MATCHED/PRESENT	Effect size r	ß	CI lower; CI upper	*p*	η^2^
**PRESENT shift workers**							
VN *within*	7.1	10.62/16.38; *p* = .057	.38	.31	− .32; .94	.290	.14
SMN *within*	9.5	13.62/13.38; *p* = .960	− .02	.19	− .58; .96	.586	.04
DAN *within*	5.9	12.23/14.77; *p* = .418	.17	− .44	− 1.18; .31	.213	.19
VAN *within*	6.5	14.85/12.15; *p* = .390	− .18	.17	− .62; .97	.628	.03
LIMN *within*	8.5	14.15/12.85; *p* = .687	− .09	− .19	− .88; .51	.558	.05
FPN *within*	8.6	**16.65/10.35; p = .034**	− .41	.06	− .70; .81	.871	.00
DMN *within*	5.0	15.08/11.92; *p* = .311	− .21	− .22	− 1.10; .67	.587	.04
VN *inter*	6.9	13.69/13.31; *p* = .920	− .03	− .53	− 1.28; .21	.138	.25
SMN *inter*	7.3	13.54/13.46; *p* = 1.000*	− .01	− .36	− 1.09; .36	.281	.14
DAN *inter*	5.0	14.00/13.00; *p* = .762	− .07	− .40	− 1.28; .48	.329	.12
VAN *inter*	7.1	15.69/11.31; *p* = .153	− .29	− .54	− 1.31; .24	.149	.24
LIMN *inter*	7.4	13.15/13.85; *p* = .840	.05	− .50	− 1.15; .16	.119	.28
FPN *inter*	8.4	15.38/11.62; *p* = .223	− .25	− .32	− 1.06; .43	.361	.11
DMN *inter*	4.8	13.62/13.38; *p* = .960	− .02	− .37	− 1.22; .49	.350	.11
VN *Ratio*	6.2	**10.46/16.54; p =.044**	.40	**.63**	**.26**;** 1.01**	**.005**	**.65**
SMN *Ratio*	5.8	13.77/13.23; *p* = .880	− .04	.30	− .47; 1.08	.390	.09
DAN *Ratio*	5.9	11.15/15.85; *p* = .125	.31	− .09	− .86; .69	.799	.01
VAN *Ratio*	5.6	13.77/13.23; *p* = .880	− .04	.40	− .46; 1.26	.312	.13
LIMN *Ratio*	6.7	14.08/12.92; *p* = .724	− .08	.22	− .49; .93	.496	.06
FPN *Ratio*	6.2	15.23/11.77; *p* = .264	− .23	.21	− .45; .87	.486	.06
DMN *Ratio*	6.5	14.15/12.85; *p* = .687	− .09	.14	− .82; 1.10	.748	.01
**FORMER shift workers**							
VN *within*	3.3	93.14/83.97; *p* = .232	− 0.09	.002	− .001; .005	.163	.023
SMN *within*	3.4	94.80/82.34; *p* = .105	− 0.12	.001	− .001; .004	.246	.016
DAN *within*	2.8	93.25/83.85; *p* = .221	− 0.09	.000	− .001; .002	.544	.004
VAN *within*	4.1	87.83/89.16; *p* = .863	0.01	− .001	− .002; .002	.978	.001
LIMN *within*	7.4	87.66/89.33; *p* = .828	0.02	.000	− .002; .001	.462	.006
FPN *within*	3.6	94.26/82.87; *p* = .138	− 0.11	.001	− .001; .002	.519	.005
DMN *within*	6.8	92.57/84.52; *p* = .294	− 0.08	.000	.000; .002	.226	.017
VN *inter*	5.0	**97.68/79.53; *p* = .018**	− 0.18	.000	− .001; .001	.938	.001
SMN *inter*	6.0	95.20/81.96; *p* = .085	− 0.13	.000	− .001; .001	.799	.001
DAN *inter*	3.0	95.22/81.93; *p* = .084	− 0.13	.000	.000; .001	.596	.003
VAN *inter*	5.7	92.54/84.55; *p* = .298	− 0.08	.000	− .001; .000	.311	.012
LIMN *inter*	4.0	95.92/81.25; *p* = .056	− 0.14	.000	− .001; .000	.834	.001
FPN *inter*	6.3	93.03/84.07; *p* = .243	− 0.09	.000	− .001; .000	.514	.005
DMN *inter*	5.2	94.99/82.16; *p* = .095	− 0.13	.002	− .001; .000	.683	.002
VN *Ratio*	5.3	86.85/90.11; *p* = .671	0.03	.002	− .001; .005	.281	.014
SMN *Ratio*	3.4	89.90/87.13; *p* = .719	− 0.03	.002	− .000; .005	.055	.043
DAN *Ratio*	4.1	89.01/88.00; *p* = .895	-0.01	.001	− .002; .003	.575	.004
VAN *Ratio*	4.1	84.53/92.38; *p* = .307	0.08	.001	− .001; .003	.307	.012
LIMN *Ratio*	5.1	86.30/90.65; *p* = .571	0.04	− .001	− .004; .002	.560	.004
FPN *Ratio*	2.9	91.26/85.80; *p* = .477	− 0.05	.002	− .001; .004	.195	.002
DMN *Ratio*	6.4	88.49/88.51; *p* = .999	0.00	.002	− .001; .004	.197	.020

Sign. = Percentage of tests that showed a significance of *p* < 0.05 when comparing PRESENT or FORMER shift workers with 1000 samples of RANDOM controls. * indicates an asymptotic significance. CI = 95% confidence interval.

Comparing mean RSFC of PRESENT shift workers to RANDOM controls, none of the tests indicated significant differences in mean RSFC between PRESENT shift workers and RANDOM controls [Table 2] under the assumption that *α* = 0.0024 (Bonferroni correction for multiple comparisons in RSFC parameters) corresponds to 99.76% of the tests showing a significant difference.

FORMER shift workers showed lower *inter-network* RSFC of the visual network (VN, *p* = 0.018) as compared to MATCHED controls [Table 2], but this difference was not significant when applying a Bonferroni-correction for multiple comparisons (*p*_*corrected*_ for RSFC = 0.0024).

The comparison between FORMER shift workers and RANDOM controls indicated that significant differences between the groups ranged from a minimum of 2.8% for *within-network*-RSFC of the dorsal-attention network (DAN) to a maximum of 7.4% for *within-network*-RSFC of the limbic network (LIMN). Thus, none of the examined parameters met the criterion of 99.76% of the tests showing a significant difference.

### Association with the number of shift work years, corrected for age, sex and education

Within PRESENT shift workers, more years of shift work were linearly associated with a higher ratio of *within- to inter-network* RSFC of the visual network (*ß* = 0.63, partial *η*^2^ = 0.65; *p* = 0.005) [Fig. 2]. Within FORMER shift workers, there was no association between number of shift work years and RSFC parameters.

None of the results regarding RSFC parameters were significant after applying a Bonferroni-correction for multiple comparisons (*p* = 0.05 divided by 21 parameters = *p*_*corrected*_ for RSFC = 0.002; Fig. 4).

### Cortical thickness

There were no differences in cortical thickness between PRESENT or FORMER shift workers and the group of all NEVER shift workers. The same was found when compared to MATCHED controls.

### Linear associations with numbers of shift work years, corrected for age, sex and education

There were no significant associations between numbers of shift work years and vertex-wise cortical thickness, neither in PRESENT nor in FORMER shift workers.

### Gray matter volume of subcortical structures

#### Analysis of variance

To examine a significant main effect of shift work on subcortical gray matter volumes we used a multivariate analysis of variance (ANOVA) using sex and shift work group with three levels (NEVER, PRESENT, FORMER) as factors, age and education as covariates and all subcortical volumes as dependent variables. Here, no significant main effect of shift work on gray matter volume of any subcortical structure was found [F (28, 1024) = 0.66, *p* = 0.913, Wilks Λ = 0.965; partial η^2^ = 0.018]. However, pairwise comparisons indicated that PRESENT shift workers had lower gray matter volumes in the left thalamus as compared to NEVER (*p* = 0.021) and FORMER shift workers (*p* = 0.050; Fig. 2), though this result was not significant when adding total gray matter volume as covariate (PRESENT < NEVER, *p* = 0.250; PRESENT < FORMER, *p* = 0.219). None of these results would be significant after post-hoc Bonferroni correction (p = 0.05 divided by 7 subcortical structures for each hemisphere = p_corrected_ for subcortical volumes = 0.05/14 = 0.0036) though.

### MATCHED samples comparisons

When compared to MATCHED controls, the difference in gray matter volume of the left thalamus of PRESENT shift workers showed trend level significance (*p* = 0.050). For all other structures Mann–Whitney-U tests did not indicate a significant difference. When FORMER shift workers were compared to MATCHED controls no significant difference was found.

### Association with numbers of shift work years, corrected for age, sex and education

A higher number of shift work years was associated with lower gray matter volume of the left thalamus for PRESENT shift workers (*ß* = − 0.613, *p* = 0.019, partial *η*^2^ = 0.516), which was also true after adding total gray matter volume as covariate (ß = −0.645, *p* = 0.016, partial *η*^2^ = 0.584, Table 3). The same was seen at trend level in FORMER shift workers (*ß* = − 0.193, *p* = 0.055, partial *η*^2^ = 0.043). When total gray matter volume was added as a covariate, the association between more shift work years and a lower gray matter volume of the left thalamus was significant (*ß* = − 0.188, *p* = 0.039, partial *η*^2^ = 0.051, Table 3).

**Table 3:** Results of subcortical structures.

Compared to	RANDOM	MATCHED		Linear regression of shift work years			
	Sign (%)	Mean rank PRESENT/MATCHED	Effect size r	ß	CI lower; upper	p	η^2^
**PRESENT shift workers**							
L Thalamus	3.50	16.46/10.54; *p* = .05	− 0.39	**− .645**	**- 50.18; **− **7.04**	**.016**	**.584**
L Caudate	3.60	13.62/13.38; *p* = .96	− 0.02	− .403	− 37.21; 13.99	.319	.141
L Putamen	4.60	13.54/13.46; *p* = 1.0	− 0.01	.434	− 51.28; 14.72	.232	.197
L Pallidum	4.10	12.69/14.31; *p* = .614	0.11	− 337	− 18.26; 7.28	.344	.128
L Hippocampus	3.90	14.00/13.00; *p* = .762	− 0.07	.142	− 18.79; 29.78	.609	.039
L Amygdala	3.50	12.54/14.46; *p* = .545	0.13	− .56	− 14.45; 11.95	.829	.007
L Accumbens	3.40	14.77/12.23;* p* = .418	− 0.17	.210	− 5.19; 2.66	.470	.077
R Thalamus	40.00	15.00/12.00; *p* = .336	− 0.20	− .262	39.51; 11.73	.241	.190
R Caudate	3.70	13.85/13.15; *p* = .840	− 0.05	− .394	− 31.25; 12.10	.331	.135
R Putamen	4.40	13.77/13.23;* p* = .880	− 0.04	− .285	− 53.32; 26.14	.445	.085
R Pallidum	16.00	11.62/15.38; *p* = .223	0.25	.551	− 20.63; 1.96	.092	.353
R Hippocampus	21.00	13.08/13.92; *p* = .801	0.06	− .061	− 15.07; 11.24	.741	.017
R Amygdala	15.00	13.00/14.00; *p* = .762	0.07	− .048	− 10.06; 8.32	.829	.007
R Accumbens	8.00	14.54/12.46; *p* = .511	− 0.14	− .287	− 8.58; 4.05	.424	.093
**FORMER shift workers**							
L Thalamus	.30	91.39/85.67; *p* = .457	− 0.06	**− .188**	− **24.92; − .67**	**.039**	**.051**
L Caudate	1.80	90.77/86.28; *p* = .559	− 0.04	− .022	− 9.53; 7.72	.835	.001
L Putamen	.80	89.35/87.67; *p* = .827	− 0.02	− .129	− 3.71; 17.20	.203	.020
L Pallidum	1.00	91.34/85.72; *p* = .464	− 0.06	.026	− 4.24; 5.57	.788	.009
L Hippocampus	0.80	90.85/86.20; *p* = .545	− 0.05	− .159	− 13.20; − 0.02	.050	.046
L Amygdala	1.00	93.14/83.96; *p* = .232	− 0.09	− .156	− 5.92; .310	.077	.038
L Accumbens	.60	93.55/83.57; *p* = .194	− 0.10	− .098	− 2.01; 1.44	.743	.029
R Thalamus	1.20	91.97/85.11; *p* = .372	− 0.07	− .076	− 18.87; 7.39	.387	.009
R Caudate	1.20	92.35/84.74; *p* = .322	− 0.07	− .051	10.34; 6.02	.600	.003
R Putamen	1.20	90.82/86.24; *p* = .551	− 0.04	.060	− 7.06; 13.13	.552	.004
R Pallidum	11.00	92.39/84.70; *p* = .317	− 0.08	.048	− 3.65; 6.02	.627	.003
R Hippocampus	11.00	90.93/86.12; *p* = .531	− 0.05	− .110	− 12.38; 2.79	.212	.019
R Amygdala	3.00	91.67/85.40; *p* = .414	− 0.06	− .143	− 5.83; .58	.107	.031
R Accumbens	1.00	92.01/85.07; *p* = .366	− 0.07	− .007	− 1.66; 1.54	.938	.000

Sign. = Percentage of tests that showed a significance of *p* < 0.05 when comparing PRESENT or FORMER shift workers with 1000 samples of RANDOM controls. * indicates an asymptotic significance. CI = 95% confidence interval. L = Left, R = Right.

### Cognitive performance

#### Analyses of variance

To examine a significant main effect of shift work on cognitive performance we used univariate analyses of variance (ANOVA) using sex and shift work group with three levels (NEVER, PRESENT, FORMER) as factors, age and education as covariates and the respective cognitive variable as dependent variable. Here, no main effect of shift work group was found [F(30, 976) = 1.03; *p* = 0.425, partial *η*^2^ = 0.031]. Pairwise comparisons indicated that FORMER shift workers performed significantly lower than NEVER shift workers (*p* = 0.017) regarding short-term memory [ZNS (Forward)]. For naming colors in the Stroop test (*p* = 0.045), pairwise comparisons indicated that FORMER shift workers were significantly slower than NEVER shift workers (*p* = 0.025).

#### Matched samples comparisons

When compared to MATCHED controls, PRESENT shift workers showed marginally faster processing speed in TMT-A (*p* = 0.044, effect size = − 0.39) and lower susceptibility to interference (Stroop, *p* = 0.039; r = − 0.41). As compared to RANDOM controls, cognitive performances of PRESENT shift workers showed no differences, as significant tests between the groups ranged from a minimum of 2.2% for short-term memory [ZNS (Forward)] to a maximum of 4.6% for processing speed (TMT-A [Table 4]).

**Table 4:** Results of cognitive performances.

Compared to	RANDOM	MATCHED		Linear regression of shift work years			
	Sign (%)	Mean rank MATCHED/PRESENT	Effect size r	ß	CI lower; upper	p	η^2^
**PRESENT shift workers**							
AKT (Time)	3.1	12.62/13.42, * p* = .810	.05	− .315	− .585; .169	.233	.195
CBT (Forward)	2.3	14.42/12.58, *p* = .139	− .13	− .298	− .070; .023	.271	.149
CBT (Backward)	3.5	12.65/14.35, *p* = .579	.12	.611	− .005; .072	.280	.474
ZNS (Forward)	2.2	13.69/13.31, *p* = .920	− .03	.044	− .044; .049	.900	.002
ZNS(Backward)	3.1	12.77/14.23, *p *= .650	.12	− .141	− .066; .044	.648	.027
TMTA	4.6	**16.50/ 10.50,** *p*** = .044**	− .39	.046	− .436; .489	.899	.002
TMTB	2.9	17.60/13.40; *p* = .202	.24	.415	− .160; .991	.137	.228
TMTBA(Switch)	2.8	11.46/15.54, *p* = .186	.27	.286	− .990; 2.372	.371	.101
Stroop(Reading)	2.7	12.31/14.69, *p* = 0.448	.16	.086	− .572; .700	.822	.007
Stroop(Naming)	4.5	11.96/15.04, *p* = .311	.20	− .098	− .593; .769	.774	.001
Stroop(Selectivity)	3.2	14.15/12.85,* p* = .687	− .09	.424	− .362; 1.403	.211	.188
Stroop(Interference)	3.2	16.62/10.38, *p* = .039	− .41	.442	− .310; 1.222	.207	.191
Reasoning	3.4	11.23/15.77, *p* = .139	.30	.032	− .340; .371	.921	.001
Visual Pattern	2.6	13.65/13.35, *p* = .92	− .03	.020	− .090; .095	.950	.001
Benton	3.1	12.50/14.5, *p* = 0511	.13	.120	− .410; .594	.684	.022
Creative Think	2.3	13.23/13.77, *p* = .880	.04	.230	− .235; .292	.538	.055
Vocabulary	3.5	11.42/14.71, *p* = .270	. 22	− .041	− .276; .244	.888	.049
**FORMER shift workers**							
AKT (Time)	4.1	83.45/93.44, *p* = .193	.10	.214	.019; .556	.036	.045
CBT (Forward)	4.0	90.95/86.11, *p* = .497	− .04	− .002	− .018; .018	.987	.001
CBT (Backward)	3.4	90.06/86.97, *p* = .674	− .02	− .065	− .031; .016	.537	.005
ZNS (Forward)	4.8	93.99/83.13, *p* = .139	− .07	− .060	− .027; .015	.575	.004
ZNS (Backward)	5.5	86.43/90.53, *p* = .549	.05	.003	− .020; .021	.977	.001
TMTA	4.3	92.57/84.52, *p* = .295	− .07	.084	− .133; .332	.397	.009
TMTB	5.0	98.37/102.64; *p* = .602	− .04	.214	− .040; .469	.098	.029
TMTBA(Switch)	5.7	88.34/88.66, *p* = .967	− .01	.141	− .293; 1.629	.170	.022
Stroop(Reading)	4.3	**80.53/96.29; ***p*** =** .040	.16	.180	− .018; .272	.084	.035
Stroop(Naming)	5.6	**80.49/96.33,** *p*** = .039**	.13	.234	.023; .450	.031	.065
Stroop(Selectivity)	3.4	86.11/90.84, *p* = .538	.03	.202	.007; 1.316	.048	.052
Stroop(Interference)	3.3	90.19/86.85, *p* = .664	.00	.180	− .069; 1.137	.082	.040
Reasoning	4.3	87.14/88.85, *p* = .823	.00	− .221	− .198; − .022	.015	.063
Visual Pattern	4.2	92.45/84.63, *p* = .301	− .05	− .076	− .046; .021	.526	.007
Benton	4.1	89.41/84.56, *p* = .524	− .05	.047	− .120; .203	.612	.003
Creative Think	4.0	90.30/85.72, *p* = .549	.01	− .127	− .276; .072	.247	.018
Vocabulary	3.4	80.53/96.29; *p* = .446	− .03	− .177	− .190; − .010	.076	.039

Sign. = Percentage of tests that showed a significance of *p* < 0.05 when comparing PRESENT or FORMER shift workers with 1000 samples of RANDOM controls. * indicates an asymptotic significance. CI = 95% confidence interval.

FORMER shift workers showed slower processing speed [Stroop (Reading; *p* = 0.040, effect size = 0.16; Stroop (naming; *p* = 0.039, effect size = 0.13)] in comparison to MATCHED controls. As compared to RANDOM controls, significant differences to FORMER shift workers ranged between a minimum of 3.3% for susceptibility to interference [Stroop(Interference)] to a maximum of 5.7% for concept shifting [TMT-BA (Switching)], thus indicating no difference between groups in cognitive performance.

### Association with numbers of shift work years, corrected for age, sex and education

In PRESENT shift workers, we observed no association between the number of shift work years and cognitive performances (Table 3) (all *p* > 0.137, all partial *η*^2^ = 0.001–0.474).

**Figure 3:**
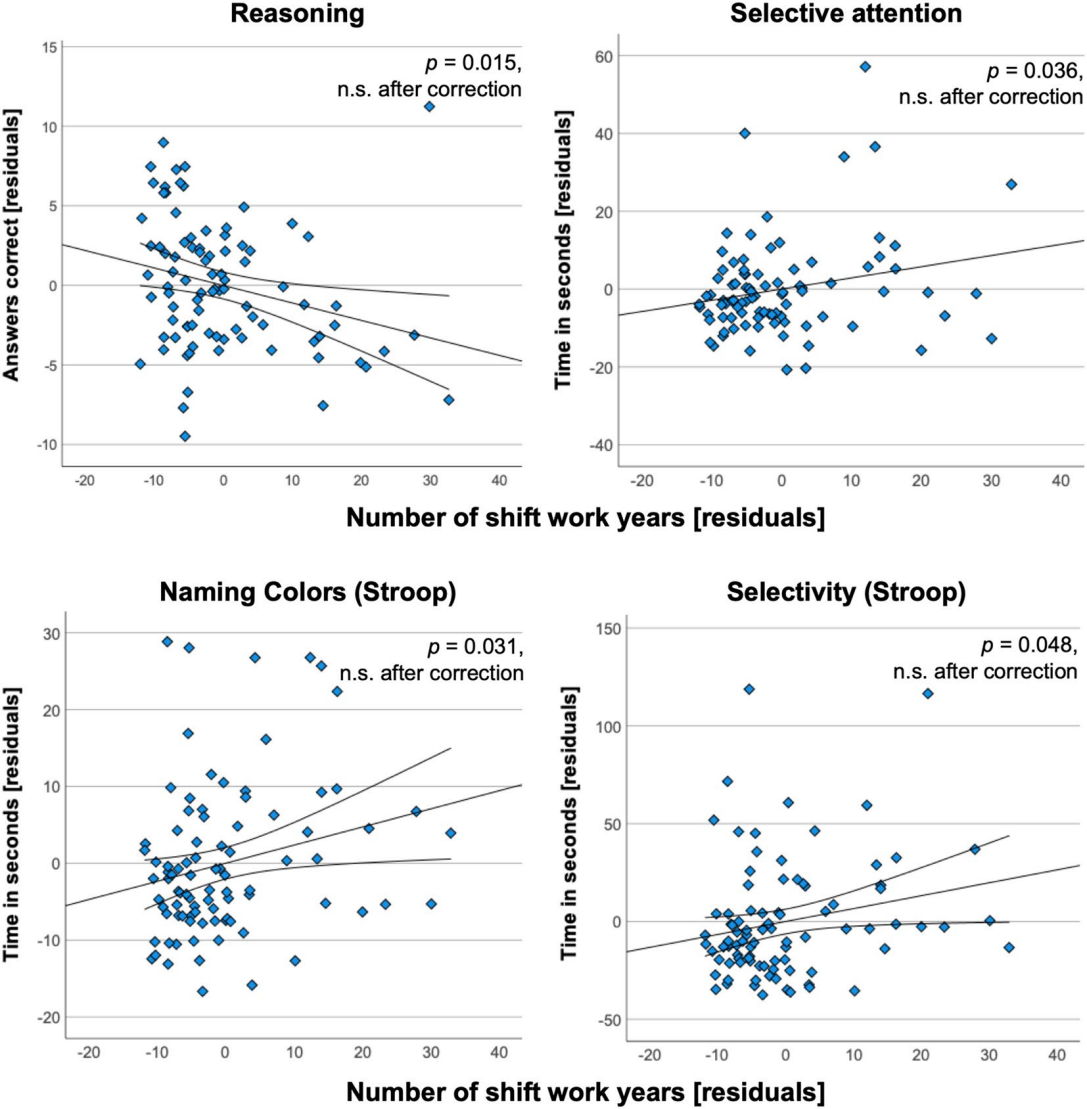
Cognitive performances. Scatter plots of partial correlations between the number of shift work years and cognitive performances in FORMER shift workers are shown. All parameters are residuals from partial correlations, corrected for age, sex and education. 95% confidence intervals are indicated by lines surrounding the regression lines and are given in detail in Table 3 for the regression coefficients. None of these assocations were significant after multiple comparison correction.

In FORMER shift workers, more years of shift work were associated with longer processing times in selective attention [*ß* = 0.214, *p* = 0.036, partial *η*^2^ = 0.045, AKT (Time)] and lower performance in reasoning [*ß* = −0.211, *p* = 0.015; partial *η*^2^ = 0.063, Fig. 3]. In the Stroop test, more years of shift work were associated with lower processing speed in naming (*ß* = 0.234, *p* = 0.031, partial *η*^2^ = 0.065) and during the selectivity task (*ß* = 0.202 *p* = 0.048, partial *η*^2^ = 0.052).

**Figure 4:**
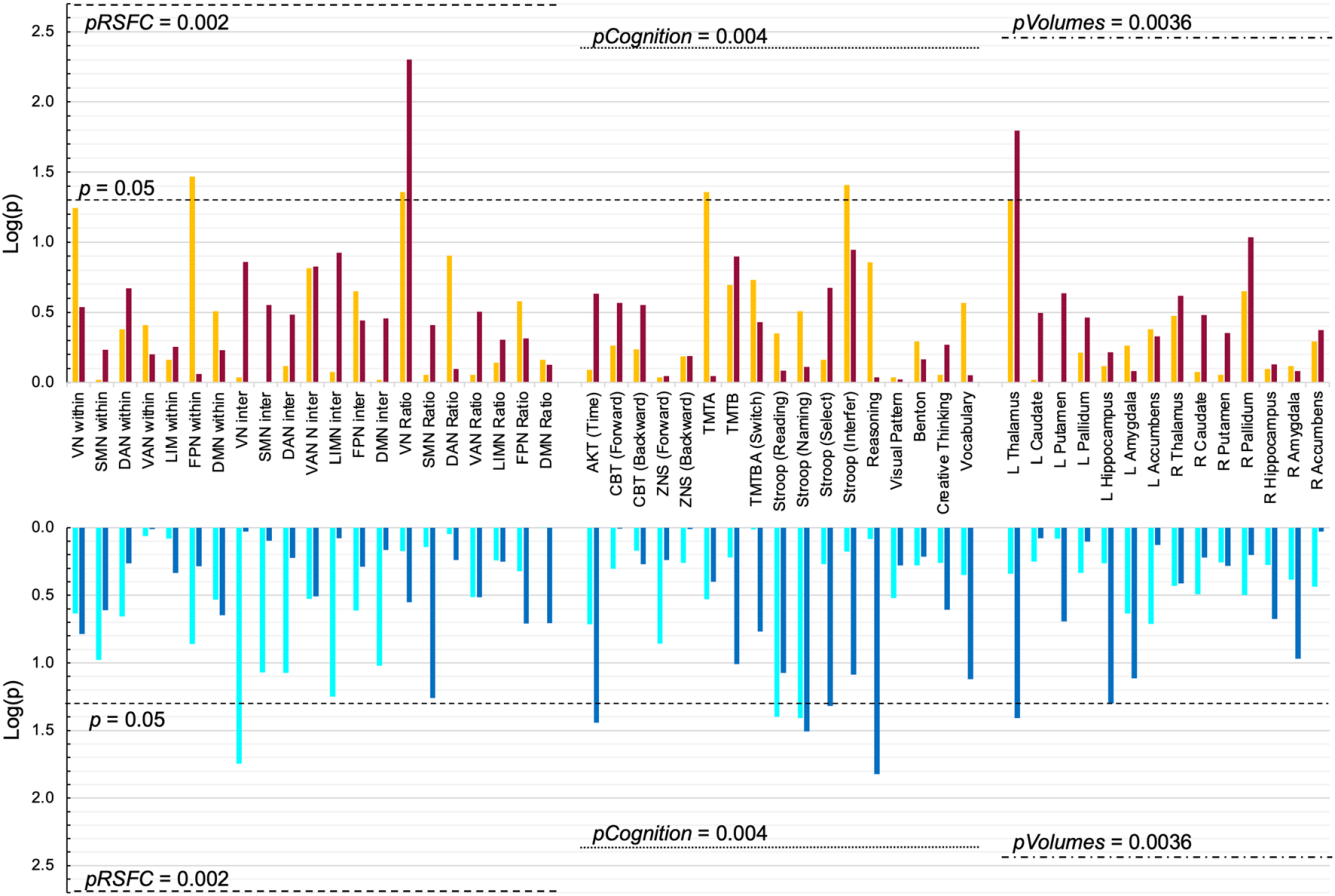
Summary of results from PRESENT and FORMER shift workers. None of the effects were significant after application of Bonferroni correction for multiple comparisons (pRSFC, pCOGNTION, pVOLUMES). Y-axis shows log-transformed p-values. (**A**) For PRESENT shift workers *p* values of comparisons to MATCHED controls are represented by orange bars. *p* values of partial correlations with the number of shift work years are represented in dark red. (**B**) For FORMER shift workers *p* values of comparisons to MATCHED controls are represented by light blue bars. *p* values of partial correlations with the number of shift work years are represented in dark blue bars. L = left, R = right.

From the effect sizes, all associations can be seen as weak associations^89^, while the confidence intervals around the regression coefficients indicate a high uncertainty since one of the intervals were close to zero (Table 4). When applying a Bonferroni-correction for multiple comparisons (*p* = 0.05 divided by 12 independent tests = *p*_*corrected*_ for cognitive performance = 0.004) none of these associations would be significant [Fig. 4].

### Mediation analyses

Partial correlations corrected for age, sex and education between all RSFC parameters and cognitive performances can be found in supplementary tables 1 to 3 for the whole sample, supplementary tables 4 to 6 for PRESENT shift workers and supplementary tables 7 to 9 for FORMER shift workers.

### Group differences

For the first series of mediation analyses, we entered shift work group (PRESENT, FORMER, NEVER) as explanatory factor (X) and cognitive performances [digit span, i.e. ZNS (Forward); Stroop (Reading; Naming); TMT-A; TMT-BA] as outcomes (Y). No mediation effect of gray matter of the left thalamus (M) was revealed.

### Shift work years

As a result of the second mediation analysis, no indirect effect of the number of shift work years via gray matter volume of the left thalamus as mediator (M) on processing speed [Stroop (reading), Stroop (naming)], selective attention or executive performance [Stroop (selectivity), Stroop (interference)] was found.

### Chronotype

Since the chronotype has been discussed to modulate RSFC^10^ as well as the ability to cope with the circadian challenges of shift work^11^, we investigated whether the three examined groups differed in terms of chronotype. Chronotype was measured as mid sleep on free days (MSF), defined as the midst between an individual´s preferred time to go to bed and to wake up, if there are no environmental restrictions^76^.

MSF of PRESENT shift workers was 03:39 [h:min] and was not significantly different from MATCHED controls with 03:37 [h:min] [Z = − 0.423, p(asymptotic) = 0.689, effect size r = 0.08).The desired wake-up time on free days of PRESENT shift workers was 28 min later (at 08:08 [h:min]) as compared to MATCHED controls, but not significant (*Z* = 0.85, *p* = 0.418, effect size *r* = 0.17. There was no significant difference in sleep duration on free days either (PRESENT = 9:04 h, MATCHED controls = 9.17 h, *Z* = − 0.14, *p* = 0.894, effect size *r* = − 0.03).

MSF of FORMER shift workers was 03:36 [h:min] and not significantly different from the MSF of MATCHED controls (3:26, h:min; Z = − 0.24; *p* = 0.813; effect size r = 0.06). Sleep duration on free days (FORMER = 8.52 h, MATCHED controls = 8:50 h; Z = − 0.40, *p* = 0.689; effect size r = 0.05), as well as wake-up time on free days (FORMER = 07:48 h:min, MATCHED controls = 07:41 h:min; Z = − 0.318, *p* = 751; effect size r = 0.05) was also not significantly different.

## Discussion

The present study tested the hypothesis whether night shift work, a challenge to the human circadian system, is associated to alterations in functional connectivity and morphological characteristics of the brain using the objective imaging methodology of MRI. These investigations were supplemented by extensive neuropsychological examinations. Moreover, the chronotype was determined for all participants. Our study revealed the following major findings: 1. The chronotype did not differ between shift workers and controls. 2. After multiple comparison correction no associations between night shift work, three graph-theoretical measures of RSFC of 7 functional brain networks, brain morphology or cognitive performances were found. 3. Before multiple comparison correction, our results hint at an association between: (i) more years of night shift work and higher segregation of the visual network in PRESENT shift workers; (ii) night shift work experience and lower gray matter volume of the left thalamus, but not cortical thickness; (iii) night shift work and lower performances in selected cognitive domains.

### Chronotype

The chronotype has to be considered as a potential modulator between shift work and cognitive performance. Early and late chronotypes are thought to differ in the strength of the circadian misalignment they experience during shift work^11^. Early chronotypes may cope better with early shifts and late chronotypes better with night shifts. E.g. in a cohort of younger participants (mean age 41.8 years), the chronotype of shift workers was later than in non-shift workers^90^. Lower performance in tasks of cognitive flexibility in shift workers was also shown to depend on the circadian phase as measured in saliva-melatonin^6^. Furthermore, a recent study on the relationship between chronotypes and RSFC reported fundamental differences in the default mode network (DMN) between early and late chronotypes^10^. These differences were considered to account for the compromised attentional performance and increased sleepiness observed in late chronotypes when extrinsic social rhythms do not match their intrinsic circadian phenotype^10^. Thus, misalignment in FC of shift workers may depend on their chronotype. However, this possibility can be ruled out in our sample, since there was no difference in chronotype between PRESENT or FORMER shift workers and matched controls.

### RSFC

To the best of our knowledge this is the first study dealing with the impact of night shift work on RSFC of the brain. The analyses of RSFC revealed no differences in 7 major networks^24,57^ considered vulnerable from prior cognitive studies between shift workers (PRESENT and FORMER) and controls after applying a Bonferroni correction for multiple comparisons. However, an association between more years of shift work and a higher segregation of the visual network was observed in PRESENT shift workers, which may be considered strong based on the effect size (partial *η*^2^ = 0.65) only. It is important, however, to address that the 95% confidence interval around the regression coefficient *ß* = 0.63 ranged from 0.26 to 1.01 showing uncertainty within this effect estimation, potentially due to the lack of power (n = 13). Further, effect sizes tend to be stronger particularly in small as compared to larger samples^86,91^. The high effect size might therefore lead to overinterpretation when generalized to the population and needs further confirmation in larger samples.

This particular effect, however, could hint at a more segregated visual network, the longer the (PRESENT) shift workers had worked in shift and therefore at a reorganization of the connectedness of the visual network with more shift work experience. Less segregation, i.e. higher integration of networks has generally been discussed as a compensational mechanism, with higher coupling being a means of supporting networks affected by structural decline (e.g. during aging) to maintain cognitive functioning^19,25^. In light of this hypothesis, we may speculate that there is less compensational effort with more years of shift work experience. High segregation of large-scale networks has also been related to better cognitive performance in healthy, young adults^25^ and supposedly reflects high specialization. *At rest* it may indicate an optimal state from which dynamic changes in connectivity can be initiated to solve a task^26^. This would fit with our observation that the visual network is more segregated with higher experience in shift work. Thus, this may be an adaptation towards the altered exogenous environment and may therefore reflect a more optimal state during rest for shift workers. Whether and how the connectivity profile of the visual network in shift workers dynamically changes during *an active state of task* needs to be elucidated by further studies.

Since no association between the number of shift work years and cognition was found for the PRESENT shift workers, we could not establish a triangular association between the number of shift work years and RSFC on the one hand and RSFC and cognition on the other hand. This may have several reasons: The first reason is the small sample size through which we lack power to find an association between shift work and cognition in PRESENT shift workers and therefore also such a triangular association. This is the most likely reason, since the association between network-wise RSFC measures and cognition is well established^25,27,59^, has previously already been shown for the here investigated cohort (please see^24^) and can be found if examined within the whole sample investigated here (Supplementary tables 1 to 3). A higher segregation of the visual network correlated also with better working memory performance [*r* = 0.81, *p* = 0.016; CBT (Backward), Supplementary table 6] in PRESENT shift workers, though this was independent of the number of shift work years. Another reason may relate to the complexity of the brain as a system: It is possible that neuronal differences related to shift work exist, but that these do not necessarily lead to differences in cognitive performance due to the huge compensatory potential of the brain^16–19^ as has often been discussed in aging research. Further, there may be no triangular association between shift work, RSFC and cognitive performance with respect to the here investigated parameters, i.e. visual network and the selected cognitive tasks. Instead, there may be other neuronal differences explaining cognitive decline. Here, other target regions may be considered, such as the thalamus (please see discussion on morphometry below) or other smaller brain circuits involved in circadian rhythms, e.g. the suprachiasmatic nucleus of the hypothalamus^92^. Further, other connectivity features may be explored in future studies, such as anatomical or task-based functional connectivity.

Since lower *inter-network* RSFC of the visual network was also observed in FORMER shift workers as compared to MATCHED controls the present results may hint at a particular role of the visual network in shift workers. In future studies with higher power, the visual-processing network may thus become an interesting target for focusing on the relationship between brain function and shift work.

### Cortical thickness and subcortical structures

We did not find any reliable association between shift work and cortical thickness at an alpha-level of 0.001, which was adjusted as suggested for surface-based analyses^83^. Thus, our results do not support an association between shift work and cortical thickness. Regarding subcortical structures, we found associations between shift work and gray matter volume of the left thalamus. Even though none of these association survived Bonferroni correction and the main effect of shift work (ANOVA) was not significant, they show a quite consistent pattern within PRESENT shift workers: Pairwise comparisons indicated that PRESENT had lower gray matter volumes than FORMER and NEVER shift workers (Fig. 2). Also, in PRESENT shift workers, a higher number of shift work years was associated with lower gray matter volume of the left thalamus. The functional role of the thalamus and its specific nuclei as modulators of circadian rhythms has been implicated in a large body of research^93,94^. Fewer studies have reported on structural alterations within the thalamus in association to circadian rhythms. Bilateral thalamic volume loss has been observed in patients with sleep insomnia^95^ and after sleep deprivation in healthy men^96^. The authors discussed this as a possible explanatory mechanism for cognitive performance reductions after sleep loss, while no explanation was given how sleep loss should cause volume loss within the thalamus. In the present study, however, the association between shift work and cognitive performance could not be explained by an indirect effect of thalamic volume loss. Additionally, only the left thalamus was affected. From the confidence intervals of the estimation of gray matter volume in PRESENT shift workers, as well as the confidence intervals around the regression slope, together with the small sample size, this effect has to be interpreted carefully. However, the association between a higher number of shift work years and less gray matter volume in the cross-sectional analysis presented here, may hint at long-term effects of shift work on one of the neuronal modulators of circadian rhythms. This has to be further examined in future studies with larger sample sizes.

In humans, there is not much research on the impact of chronodisruption despite sleep disorders and deprivation on morphological brain characteristics and, to the best of our knowledge, no study on the impact of shift work on brain morphology. Cho^39^ investigated the impact of chronic jetlag on the volume of the right temporal lobe of flight attendants. In those with short, but not long recovery periods a correlation was found between saliva cortisol levels, lower volume of the right temporal lobe and longer reaction times in a visual-spatial memory task^39^. In the present study no correlation was found between shift work and the temporal lobe, neither within the surface-based analysis of the whole cortex nor within the hippocampus (analysis of subcortical gray matter volumes). This may be due to the different kind of chronodisruption (jetlag versus shift work) or to methodological reasons, e.g. that we chose a whole-surface versus region-based approach and an older while Cho^39^ investigated a younger sample.

### Cognitive performances

The present results do not support a general association between shift work and cognitive ability, because only some tests indicated differences between shift working groups (Fig. 3). Of the large battery of cognitive tests used here, the Stroop test was the most sensitive test. Here, our results mostly hinted at lower performances in PRESENT and FORMER shift workers (ANOVA & MATCHED analysis). A higher number of shift work years was also correlated to longer processing times in FORMER shift workers (Fig. 3). Therefore, our results might hint at an association between shift work and specific parameters of lower processing speed and cognitive flexibility. Nevertheless, these correlations were not significant after multiple comparison correction.

Previous studies reported associations between shift work and cognitive performance in varying parameters but with variable and inconsistent results as outlined in the introduction. After night shift, lower performance in tasks of cognitive flexibility was described, but this depended on the circadian phase determined by melatonin levels in saliva^6^ and was associated to sleepiness. Unfortunately, no control group was included in this study and thus no information was provided whether the overall performance was lower in shift workers than in non-shift workers. In a large epidemiological study, present but not former shift workers, showed slower performance in all three subtasks of the TMT than never shift workers^8^, which fits with the results from our study. Here, PRESENT shift workers showed faster reaction times as compared to MATCHED controls in the processing speed measure of TMTA. No correlations with the number of shift work years were found. In another study, night shift workers made more errors, but reaction times in working memory, sustained attention and processing speed measured with the TMT were comparable to day shift workers^2^, thus whether an effect is found may also largely depend on the parameter investigated (reaction times versus correct answers given). Performance of emergency physicians was comparable after overnight shifts and dayshift, but working memory seemed to be slightly impaired after night shift^5^. Simulated night shifts seem to impair vigilance and cognitive control^3^. Even within the attention system subprocesses (orienting versus alerting) rely differentially on time of day and chronotype^36^. These studies show that disruptions of the intrinsic circadian rhythm do not affect global cognitive performance, but rather specific cognitive processes, as is also suggested by our study. On the other hand Marquié, et al.^4^ found worse performance in current shift workers in a global cognitive score. Taken together these studies clearly point toward the complexity of the association between shift work and cognitive performance. This may be attributed to other influencing factors, such as the time, at which cognitive performance is measured, e.g. directly after the end of shift, or the recency of shift work, i.e. after retiring the effects seem to vanish^4^, but also the specific shift work phenotype. E.g. slightly impaired cognition in later life of former shift working nurses was found only, if they had a shift work history of more than 10 years^4^ or 20 years^7^. In the current study, decreases in cognitive performances were also found with a higher number of shift work years. Even though the present correlations were not significant after multiple comparison correction, it may be inferred that the number of shift years has to be considered as a factor influencing cognitive performance.

Another factor that may play a role, particularly when it comes to neuronal differences associated to circadian disruptions may be elevated stress^39,97^. This is emphasized in studies showing that no disruption in cognitive performance was found in shift workers who were able to adapt to their working schedule^6,8^. Thus, the individual ability to cope with exogenous influences on circadian rhythms and individually perceived psychological stress may be important influences to be investigated in future studies with potentially greater power.

One limitation of the present study is the rather limited number of PRESENT shift workers. Therefore, all effects reported and discussed here should be regarded with a respective uncertainty, but they provide valuable hints towards interesting targets for the future assessment of neuronal differences related to shift work. It is also important to keep in mind that the 1000BRAINS cohort, on which the present study was built, is a population-based cohort. Future studies may collect objective imaging data in populations of shift workers as has been done with cognitive data^7,98^. One clear advantage of the present study is the rich multimodal imaging data available in the 1000BRAINS cohort.

## Conclusion

In summary, no associations between night shift work, three graph-theoretical measures of RSFC of 7 functional brain networks and brain morphology were found after multiple comparison correction. Preceding multiple comparison correction, our results hinted at an association between more years of shift work and higher segregation of the visual network in PRESENT shift workers, as well as lower gray matter volume of the left thalamus. Extensive neuropsychological investigations supplementing objective imaging methodology did not reveal an association between night shift work and cognition after multiple comparison correction. Our pilot study suggests that night shift work does not elicit general alterations in brain networks and affects the brain only to a limited extent. These results now need to be corroborated in studies with larger numbers of participants.

Even though the sample of PRESENT shift workers was small and the absence of associations may also be attributed to limited power (n = 13) this study can be considered as a pioneer project to conduct deeper research into the neuronal basis of the association between shift work and (cognitive) health. It is expected that the future application of imaging-based objective methods in greater sample sizes will greatly contribute to evaluate the impact of perceived stress^97^ and the specific phenotype of shift work, e.g. recovery periods^39^ and organization plans.

## Supplementary Information

Supplementary Information.

## Data Availability

The datasets generated and/or analyzed during the current study will be made available from the corresponding author to other scientists on request in anonymized format and according to data protection policy in the ethics agreement.
